# Supplementary material for: Transnuclear mice reveal Peyer's patch iNKT cells that regulate B‐cell class switching to IgG1
Source: EMBO J. 2019 May 31;38(14):e101260. doi: 10.15252/embj.2018101260 (PMC6627243; doi:10.15252/embj.2018101260)
Supplement: Supplementary file 1 — Appendix [file EMBJ-38-e101260-s001.pdf]

Appendix

Table of contents:

Figure legend and reference Page 1
Figure S1 Page 2

Figure S1. PP-NKT are not enriched for gene signatures consistent with NKT0, NKT1, NKT2, NKT17, or adipose NKT cells. (A) Expression levels of signature genes associated with the indicated NKT subsets. FPKM values are from bulk RNAseq of sorted spleen or PP-NKT populations from Vα14 TN mice. Values are means of 3 biological replicates; error bars are SEM. (B) Heatmap showing expression of gene sets associated with NKT0, NKT1, NKT2, or NKT17 cells as reported in (Engel, Seumois et al., 2016).

Reference:

Engel I, Seumois G, Chavez L, Samaniego-Castruita D, White B, Chawla A, Mock D, Vijayanand P, Kronenberg M (2016) Innate-like functions of natural killer T cell subsets result from highly divergent gene programs. *Nat Immunol* 17: 728-39

A

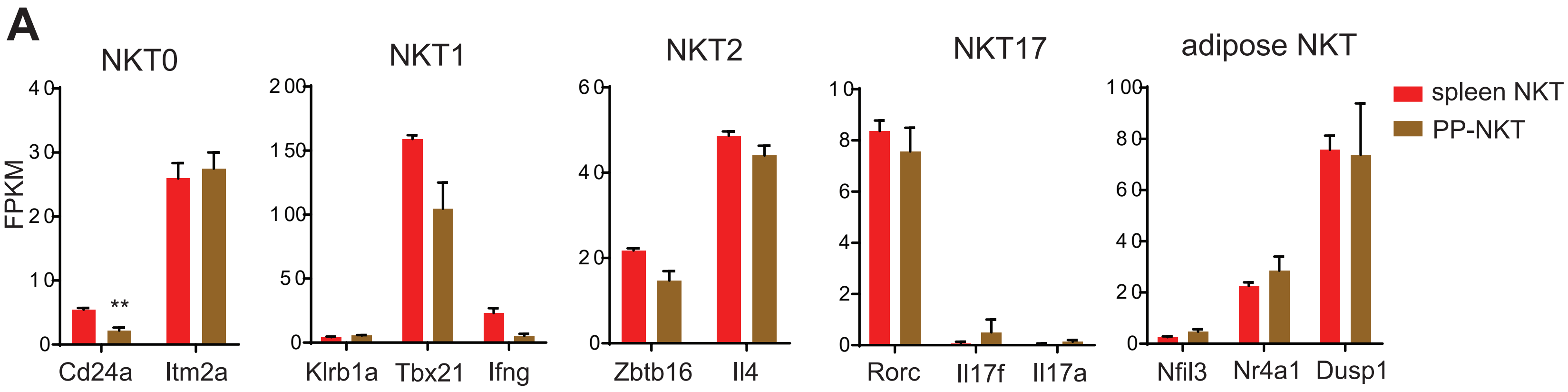

B

NKT1	spleen NKT			PP-NKT		
Nkg7	879.6	1167	886.1	1281	1504	1517
Klrd1	80.7	86.8	73.0	43.4	56.9	48.1
Ifitm10	7.8	10.0	8.2	5.4	4.9	3.0
Ly6c2	377.0	305.8	303.1	140.9	117.1	128.6
Klrb1c	106.7	150.2	129.4	84.4	121.5	74.2
Klrk1	63.7	70.0	65.3	26.5	23.3	14.9
Xcl1	110.6	119.3	113.8	113.9	130.7	104.0
Fasl	46.0	49.4	51.6	52.1	67.3	59.4
Gzmb	14.6	7.4	4.5	46.1	61.0	211.2
Cxcr3	287.8	321.5	330.6	245.2	308.2	233.7
Gimap3	815.2	660.4	834.0	1091	1027	1148
Klra3	7.9	10.6	8.0	2.6	4.4	1.4
Ms4a4b	1349	1171	1098	1356	1160	1328
Klra9	3.9	5.4	2.9	0.1	1.1	1.9
AW112010	1359	1338	1274	1576	1655	1879
Slamf7	20.4	24.7	23.7	16.4	22.3	12.7
Klrc2	22.7	22.5	19.3	5.9	5.3	3.5
Lrrk1	2.9	3.5	3.7	1.6	2.7	1.7
H2-Q7	182.1	174.1	142.9	165.8	177.4	159.4
Il2rb	432.4	442.4	470.6	303.4	446.8	267.4
Rgs1	36.4	37.1	37.2	63.2	85.3	61.7
Fcer1g	66.7	66.9	56.9	29.3	30.6	41.7
Hsd11b1	97.4	85.9	95.9	97.8	70.3	107.4
H2-Q6	271.8	222.4	230.8	296.1	295.3	272.0
Dopey2	6.9	6.1	6.4	4.0	6.0	4.3
Stat4	84.2	78.5	89.6	101.8	115.9	92.5
Fgl2	13.9	16.1	19.1	17.3	26.3	26.5
Klre1	15.4	15.4	8.0	2.9	4.5	3.2
Ctla2a	43.6	51.7	39.3	26.2	22.9	34.1
Klra5	0.1	0.5	0.2	0.4	0.0	0.1
Klrc1	96.3	124.7	92.5	23.5	24.5	10.6
Dapk2	3.0	3.9	4.5	52.3	41.3	60.9
Ppm1j	19.4	16.6	21.7	16.2	16.0	12.2
Styk1	0.0	0.0	0.1	0.0	0.0	0.0
Klra13-ps	0.3	1.1	0.3	0.1	0.0	0.1
Il12rb2	15.1	16.0	15.8	10.8	16.3	8.7
Itga1	0.9	1.2	0.9	2.5	3.0	1.9
H2-K1	2351	1993	2142	2738	2382	2830
Pik3ap1	11.7	14.3	14.9	10.2	16.3	6.7
Klra8	0.5	0.6	0.3	0.0	0.0	0.0
Klra6	1.2	0.7	0.6	0.4	0.0	0.6
Klra14-ps	0.3	0.0	0.1	0.0	0.0	0.0
Gm8909	45.6	41.4	41.6	57.2	48.3	58.9
Rps27	779.1	1091	618.1	285.7	477.3	543.5
Klra10	0.5	0.6	0.7	0.2	0.2	0.1

NKT2	spleen NKT			PP-NKT		
Plac8	307.6	269.4	229.6	684.4	857.2	917.9
Phgdh	72.1	90.1	70.1	73.0	66.5	68.8
Il17rb	39.0	38.3	36.7	35.9	40.9	34.8
2810417H13Rik	21.6	35.0	20.5	3.8	3.1	4.1
Tesc	112.5	93.2	99.9	73.7	63.7	83.5
Tyms	6.1	9.1	5.5	2.6	2.1	2.4
Asf1b	31.7	45.4	31.1	15.2	11.4	15.2
Anp32e	71.7	96.2	79.0	47.9	62.5	44.1
Phf11b	171.8	95.5	114.2	204.0	152.3	159.6
Il4	46.8	48.7	50.4	40.4	48.3	43.3
Psmg2	65.4	71.4	61.3	55.8	51.7	57.6
Cenpa	38.9	43.7	33.3	19.0	14.2	18.4
Drosha	8.1	10.2	10.0	6.2	8.9	8.8
Zbtb16	20.9	22.2	22.3	14.0	18.8	11.4
Tkt	83.0	94.6	80.2	101.2	82.5	93.0
Rrm1	28.5	40.9	34.2	26.9	22.9	20.0
Birc5	38.9	54.8	32.7	6.2	4.4	6.2
Rrm2	45.9	61.3	41.3	8.3	7.0	9.2
Cdca8	17.5	26.8	14.9	5.7	4.0	4.1
Top2a	14.2	18.5	12.6	2.5	2.1	2.0
Pmf1	44.7	50.7	40.8	38.2	32.4	44.9
Impa2	15.7	17.8	13.1	11.6	8.8	15.9
Ccnb2	25.5	31.9	19.6	3.6	3.0	2.2
Ccnb1	14.6	18.1	10.5	2.7	2.1	3.0
Cdk1	6.7	10.0	6.0	1.8	1.0	1.2
Tacc3	16.2	19.4	13.0	6.4	5.6	7.2
Igfbp4	243.4	175.8	203.7	270.2	197.2	296.2
Tk1	11.9	14.5	8.6	2.4	2.2	1.4
Racgap1	17.6	20.2	15.6	10.8	8.2	8.5
Gmnn	24.2	22.6	16.2	14.2	13.7	9.7
Dfna5	1.0	0.7	0.9	0.7	0.6	0.7
Ncaph	8.2	13.7	8.5	2.5	2.3	2.5
Aurkb	11.2	17.7	9.4	3.6	0.6	2.6
Ccne1	7.7	9.0	5.9	3.7	2.4	1.7
Hist1h2ae	32.9	43.4	25.2	3.6	2.9	4.5
Chdh	5.8	9.4	8.5	4.5	7.4	4.3
Fbln1	0.8	0.6	1.1	0.1	0.3	0.4

NKT0						
Itm2a	21.7	30.1	26.0	32.5	24.2	25.7
Ldhb	60.9	65.9	57.8	55.7	49.6	64.3
Lef1	226.7	173.5	206.5	280.5	198.9	259.0
Cd2	414.7	359.3	384.1	410.3	288.4	418.7
Ccr9	67.1	57.0	64.4	132.9	160.4	138.4
Gsn	33.9	33.7	32.6	51.0	39.7	58.1
Tspan13	138.5	112.8	136.5	227.9	198.0	263.3
Lztfl1	14.5	13.2	12.5	26.5	21.1	26.7
Zcchc12	0.5	0.2	0.7	1.8	1.4	1.7
Tnfrsf4	0.5	0.4	0.4	2.2	0.6	0.9
Cd24a	5.9	4.9	5.5	1.9	1.5	3.1
Gpr83	0.5	0.2	0.7	1.4	1.0	0.4

NKT17	spleen NKT			PP-NKT		
Rorc	8.5	9.0	7.6	6.9	9.4	6.4
Tmem176a	35.0	29.7	26.7	59.8	69.0	65.9
Serpinb1a	17.2	20.8	15.9	45.0	34.7	46.6
Blk	7.1	4.8	5.5	6.1	4.5	5.8
Tmem176b	50.2	48.5	34.6	98.1	97.2	104.6
Pxdc1	14.3	14.5	11.2	15.3	10.9	18.2
S100a4	310.4	266.5	213.9	55.1	41.1	37.5
Actn2	2.3	2.3	2.5	5.4	4.2	5.4
Il1r1	0.5	0.7	0.2	0.5	1.1	0.5
Il23r	2.5	4.3	2.1	8.1	5.2	6.0
Ccr6	6.4	7.0	7.2	3.5	3.8	3.6
5430421N21Rik	3.9	4.3	5.9	6.4	9.8	7.9
Aqp3	14.1	25.0	20.1	19.4	23.9	16.7
Sepp1	87.7	85.2	98.8	110.8	100.8	119.1
Lrrc17	0.3	0.3	0.1	0.4	0.1	0.3
Tuba8	1.0	0.3	0.2	2.3	3.5	4.1
Il17re	3.7	6.6	2.6	3.6	3.4	6.5
Cabin1	9.7	8.8	10.1	11.5	11.4	9.4
Amica1	12.8	11.8	8.0	29.1	28.4	33.7
Cd7	370.8	347.8	312.7	725.9	517.5	758.1
Chad	2.5	2.9	3.7	7.4	8.9	7.9
Apol7b	45.2	44.4	50.9	107.4	112.9	116.4
Tnfrsf25	107.5	76.3	92.5	100.1	76.8	96.2
Stab2	0.1	0.1	0.1	0.0	0.1	0.0
Emb	198.7	176.1	181.5	259.9	230.0	293.4
Sdc1	6.7	7.3	6.7	5.9	6.6	4.6
Itgae	12.6	12.0	11.4	66.3	73.0	74.1
Kcnk1	1.3	1.2	1.1	1.8	2.4	0.7
Cxcr6	854	1003	996	846	849	856
Avpi1	7.3	7.6	6.2	8.5	5.5	12.4
Prelid2	4.5	7.3	5.5	5.6	9.9	10.0
Ramp3	11.9	12.4	11.7	28.6	19.0	39.0
Gpr114	31.7	35.2	29.6	56.3	70.8	65.4
5830411N06Rik	26.2	31.7	22.5	23.7	24.1	24.5
Plekhf1	17.5	20.0	18.4	22.4	29.3	18.6
Abhd15	8.7	9.3	10.3	12.7	16.1	15.0
Abi3bp	0.1	0.1	0.2	0.1	0.1	0.1
2310007L24Rik	0.9	0.4	0.7	3.0	2.2	2.1
Jag1	0.2	0.2	0.4	0.1	0.1	0.1
Mycn	1.4	1.1	0.7	0.1	0.1	0.1
Npl	0.8	0.2	0.6	0.4	0.7	1.6
Rnf208	0.3	0.2	0.3	0.9	2.0	2.0
Mmp25	1.1	0.6	1.3	1.1	1.4	0.5

Appendix Figure S1
