# Supplementary material for: Transnuclear mice reveal Peyer's patch iNKT cells that regulate B‐cell class switching to IgG1
Source: EMBO J. 2019 May 31;38(14):e101260. doi: 10.15252/embj.2018101260 (PMC6627243; doi:10.15252/embj.2018101260)
Supplement: Supplementary file 2 — Expanded View Figures PDF [file EMBJ-38-e101260-s002.pdf]

Expanded View Figures

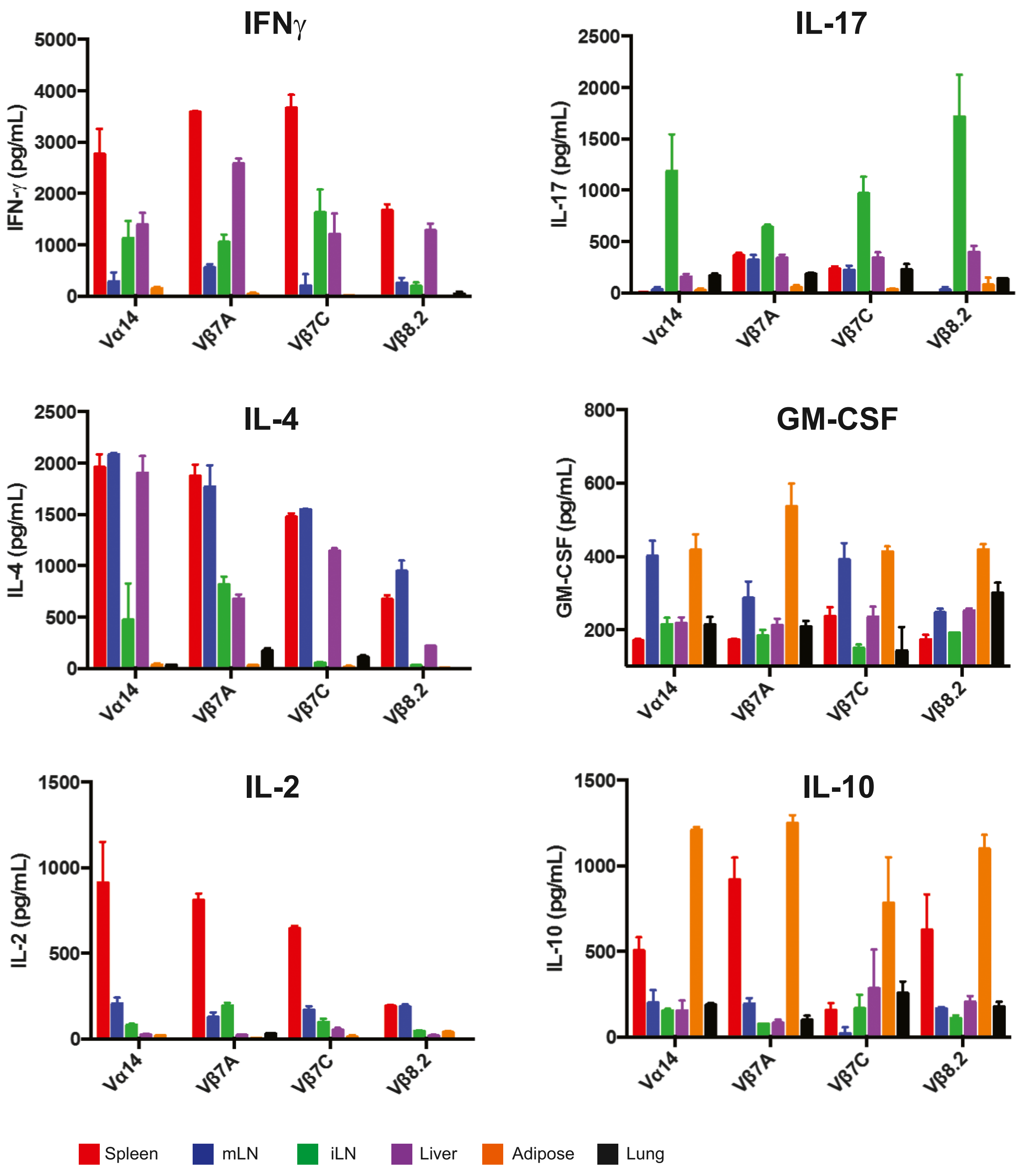

Figure EV1. Cytokine production is influenced by tissue microenvironment, not by TCR specificity.
Spleen, mLN, sdLN, liver, and adipose iNKT cells from Vα14, Vβ7A, Vβ7C, and Vβ8.2 mice (n = 3 mice per group) were stimulated *in vitro* with RAW-CD1d cells and 1 μg α-GalCer. Supernatants were collected after 24 h and cytokine concentrations determined by cytokine bead array. Error bars show SD of mean values. Results shown are representative of three independent experiments where n = 3 biological replicates per experiment.

 © 2019 The Authors

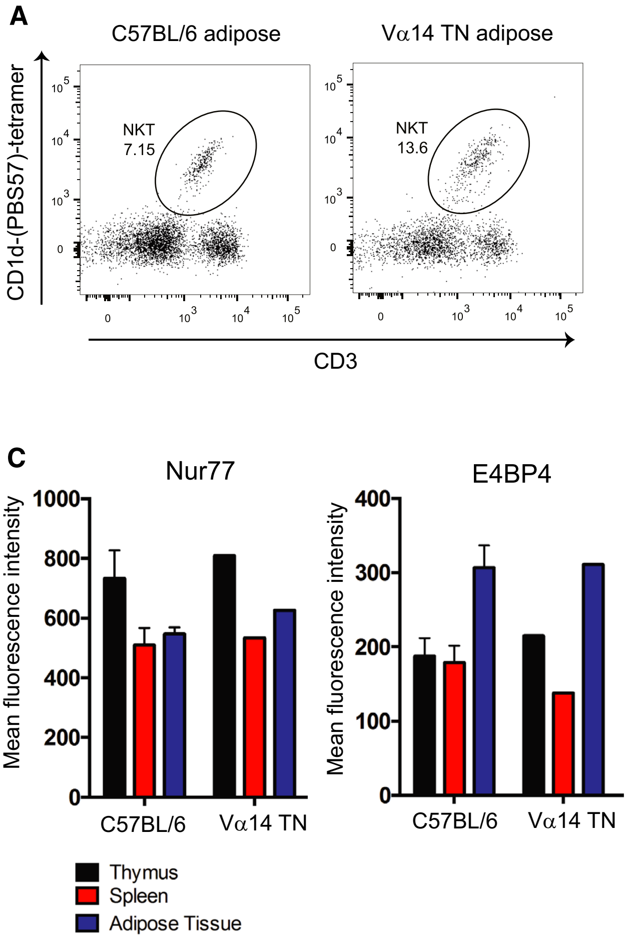

Figure EV2. Adipose iNKT cells from Vα14 TN mice are indistinguishable from C57BL/6-derived adipose iNKT cells.

A Flow cytometry analysis of iNKT cell abundance in white adipose tissue from a Vα14 TN mouse.

B Spleen cells and stromal vascular fractions of white adipose tissue from Vα14 TN or C57BL/6 mice were stained intracellularly with anti-PLZF and analyzed by flow cytometry. Histograms shown are gated on CD1d-(PBS57)-tetramer^{+} CD3^{+} cells.

C Thymus, spleen, and adipose tissue were harvested from C57BL/6 mice and Vα14 TN mice. Cell suspensions were stained with antibodies to CD3, Nur77, E4BP4, and CD1d-(PBS57)-tetramer and analyzed by flow cytometry. Mean fluorescence intensity of Nur77 and E4BP4 staining after gating on iNKT cells is shown. N = 3 per group. Error bars are SEM.

D Representative histograms of E4BP4 staining are shown. Plots are gated on total CD3^{+} cells.

© 2019 The Authors

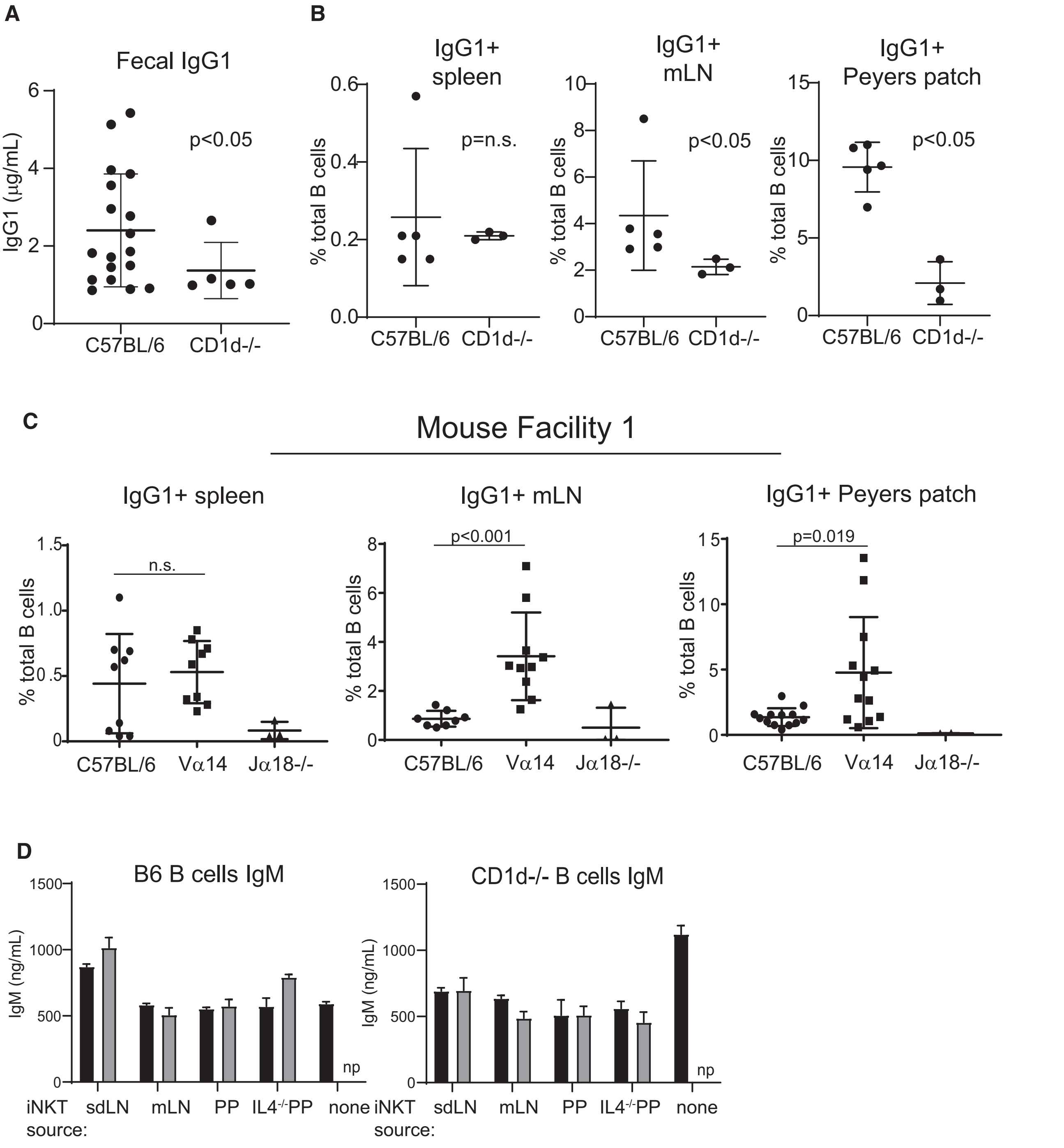

Figure EV3. Magnitude of regulation of IgG1⁺ B cells by NKT cells varies by mouse facility, but is dependent on CD1d.

A Stool collected from C57BL/6 and $CD1d^{-/-}$ mice was analyzed by ELISA for IgG1 as described in Fig 3. Mice were age- and sex-matched and housed in the Longwood Center facility. C57BL/6 $n = 18$; $CD1d^{-/-}$ $n = 5$. Mann–Whitney test. Error bars are SD.

B Spleen, mLN, and PP cells were harvested from wild-type or $CD1d^{-/-}$ mice, stained with antibodies to B220 and IgG1, and analyzed by flow cytometry. Mice were age- and sex-matched and housed in the Longwood Center facility. C57BL/6 $n = 5$; $CD1d^{-/-}$ $n = 3$. Mann–Whitney test. Error bars are SD.

C Analysis was performed identically to that shown in Fig 3F, except that mice were housed in the Smith Building at Dana-Farber Cancer Institute prior to moving to the Longwood Center at Dana-Farber Cancer Institute. Results in Fig 3 are entirely from mice housed in the Longwood Center facility. C57BL/6 $n = 10$; $V\alpha14^{-/-}$ $n = 12$; $J\alpha18^{-/-}$ $n = 3$. Mann–Whitney test. Error bars are SD.

D Culture supernatants from Fig 5D and E were measured by ELISA for IgM. np = not performed. Mann–Whitney test. Error bars are SD.

 © 2019 The Authors

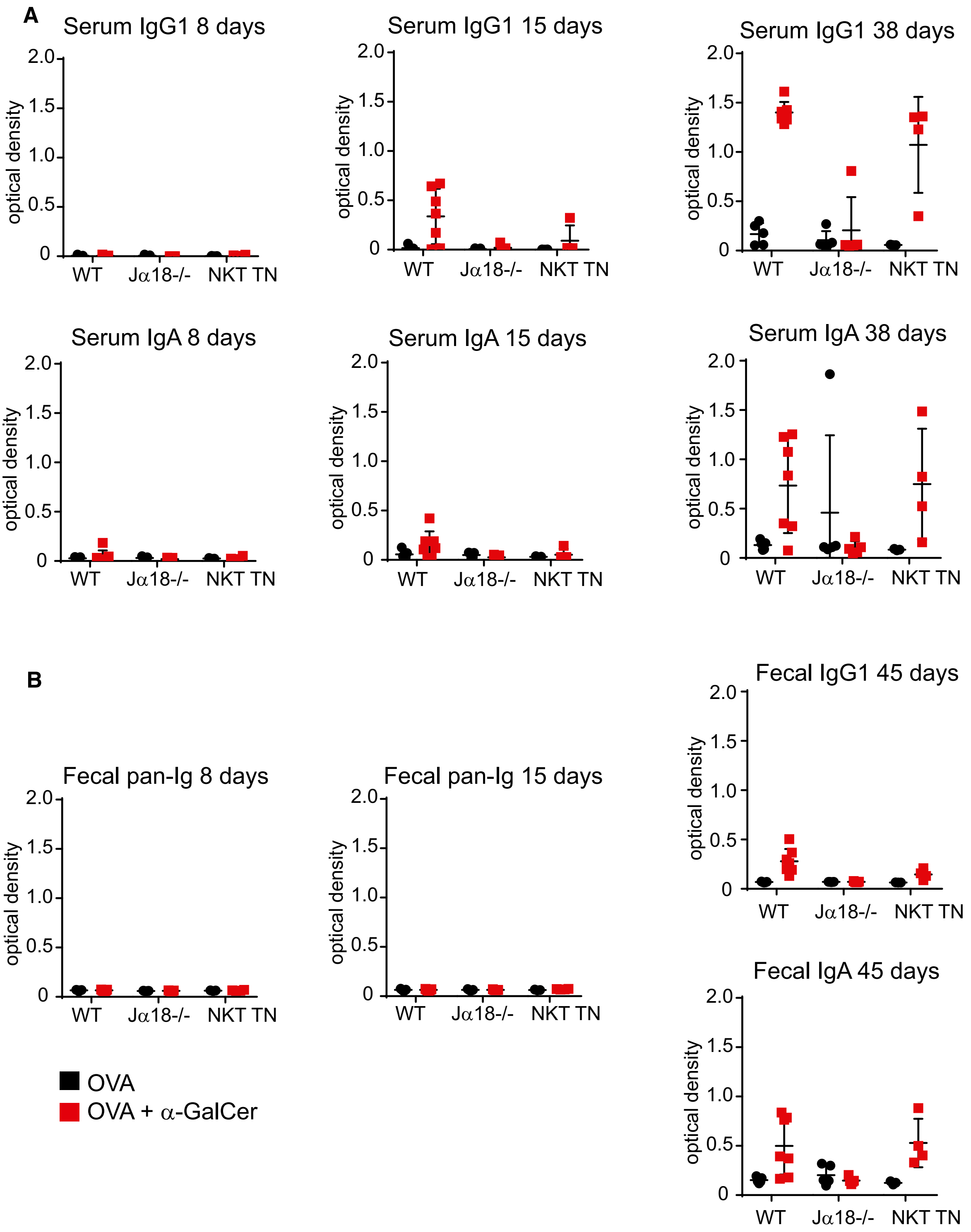

Figure EV4. Vaccination data from earlier time points for oral vaccination shown in Fig 5.

A Anti-ovalbumin antibodies were detected by ELISA from serum of immunized mice as indicated. Secondary antibodies to either IgG1 or IgA were used for detection.

B Fecal pellets were collected from immunized mice and diluted in water at 10 mg stool/ml. Anti-ovalbumin antibodies were detected by ELISA as indicated. Secondary antibodies to pan-Ig, IgG1, or IgA were used for detection.

Data information: Error bars are SD. Mice used were Jα$18^{-/-}$ (*n* = 10 total), iNKT transnuclear (*n* = 7 total), or littermate controls of iNKT TN mice that did not inherit the Vα14 allele (B6, *n* = 12 total).

© 2019 The Authors

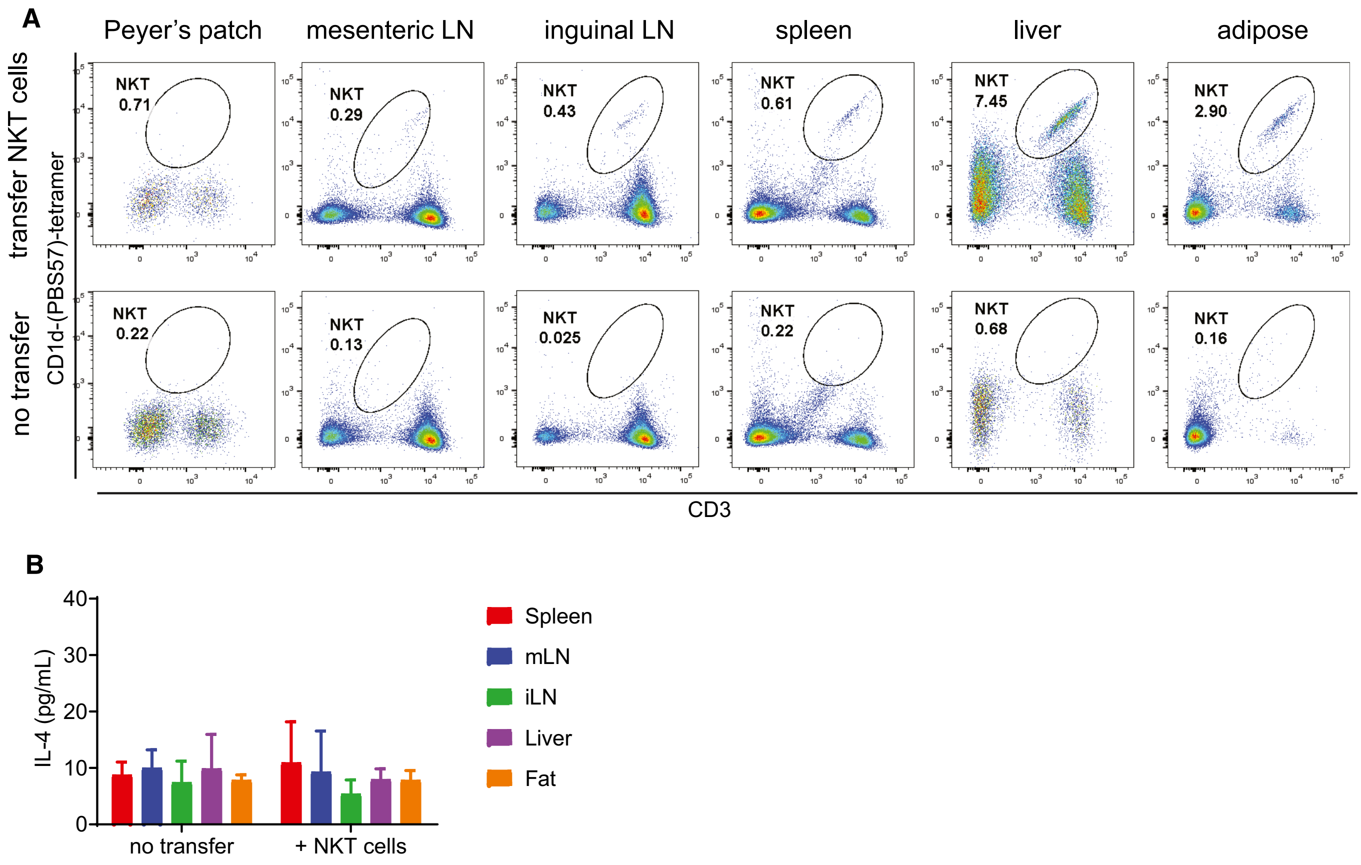

Figure EV5. Adoptively transferred iNKT cells do not accumulate in Peyer's patches and do not produce IL-4.

A $J\alpha18^{-/-}$ mice were lightly irradiated with 100 Gy prior to receiving either no cell transfer (bottom panels) or transfer of 10 million pooled spleen and LN cells from Va14 TN mice. Input cells were verified by flow cytometry to contain > 30% CD1d-tetramer^{+} cells. Two weeks post-transfer, the indicated tissues were harvested and analyzed by flow cytometry. Representative of n = 5 recipient mice per group. Representative of three independent experiments.

B Lymphocyte preparations from the indicated tissues of $J\alpha18^{-/-}$ mice from (A) were cocultured with RAWd cells and 1 μg α-GalCer as in Fig 1. IL-4 was measured by ELISA of 48-h culture supernatants. Error bars are SD of triplicate samples.

 © 2019 The Authors
